# Fermented or Floral? Developing a Generalized Food Bait Lure to Monitor Cutworm and Armyworm Moths (Lepidoptera: Noctuidae) in Field Crops

**DOI:** 10.3390/insects14020106

**Published:** 2023-01-19

**Authors:** Ronald E. Batallas, Maya L. Evenden

**Affiliations:** Department of Biological Sciences, University of Alberta, CW-405 Biological Sciences Building, Edmonton, AB T6G 2E9, Canada

**Keywords:** cutworm, armyworm, *Euxoa ochrogaster*, semiochemical, acetic acid, alcohol, 3-methyl-1-butanol, 2-methyl-1-propanol, floral volatile, phenylacetaldehyde, electroantennogram

## Abstract

**Simple Summary:**

Cutworms and armyworms are part of a group of moth pests that cause occasional damage to field crops on the Canadian Prairies. There are no reliable tools for monitoring the moth population before larval damage occurs. Food bait volatiles attract male and female moths and could be used to monitor several pests with a single lure in a single trap. We focused on enhancing a food bait lure of acetic acid and isoamyl alcohol to monitor the redbacked cutworm and other moths in canola and wheat fields. Trapping experiments tested food bait at different release rates, from different dispensers and in combination with various chemicals. High-release lures captured more females in canola, while low-release lures captured more males in wheat. Therefore, the crop where traps are positioned affects the moth trap catch. Food bait combined in an inert matrix caught more moths than bottles or plastic bag dispensers did. More females were attracted to food bait lures with isobutanol than to those with floral volatile. Fermented volatiles appear to be a more reliable attractant than floral volatiles for these moths. Depending on the sex, the feeding status affected the strength of the antenna response to the different volatiles. Females had a higher response to isobutanol if the moth was previously fed a sugar solution. Food bait lures should be further developed to monitor redbacked cutworm moths and other pests in field crops.

**Abstract:**

Cutworms and armyworms (Lepidoptera: Noctuidae) are a pest complex in North America that cause sporadic damage in field crops on the Canadian Prairies; however, no methods have been developed to reliably monitor population densities. Food-based semiochemicals attract both sexes of adult moths and could be used to monitor multiple species with a single lure in a single trap. Here, we focus on enhancing the attractiveness of acetic acid and 3-methyl-1-butanol (AAMB) lures to redbacked cutworm (*Euxoa ochrogaster*) (RBC) and other noctuid pests. Experiments conducted in canola and wheat fields tested AAMB lures at different release rates, from different devices and in combination with other semiochemicals. High-release lures captured more females in canola, while low-release lures captured more males in wheat. Thus, crop volatiles may influence response to lures. Semiochemicals embedded in an inert matrix caught more RBC moths than semiochemicals released from Nalgene or polyethylene dispensers did. More RBC females were attracted to AAMB lures with 2-methyl-1-propanol than phenylacetaldehyde. Fermented volatiles appear to be a more reliable attractant than floral volatiles for these species. RBC moth antennae produced significant responses to all doses of phenylacetaldehyde tested in electroantennogram assays, but only to higher doses of acetic acid and 3-methyl-1-butanol. Physiological state of the RBC moths also influenced responsiveness to the tested semiochemical. Feeding status did not influence the antennal response to acetic acid and phenylacetaldehyde in either sex, but it increased the response to 3-methyl-1-butanol in females when fed. AAMB lures should be further developed to monitor RBC moths and other noctuid pests in field crops.

## 1. Introduction

Cutworms and armyworms (Lepidoptera: Noctuidae) are part of a pest complex in North America that cause sporadic economic damage to annual field crops [1]. Both larvae and adults are generalist herbivores on a wide range of host plants in several different families [2]. Cutworm injury generally occurs in early summer. Late instar larvae display the characteristic cutworm feeding behaviour and cut seedling stems to feed on the stem and foliage that ultimately kills the plant [2]. In contrast, armyworm injury generally occurs in mid to late summer. Late instar larvae feed on foliage and can disperse en masse across the landscape in search of host plants when food is depleted [3,4]. Larval feeding at low population densities results in crop thinning; however, outbreaks can cause complete destruction of fields and yield loss [2].

Adult cutworms and armyworms are large, robust-bodied moths that are able to disperse over long distances [5,6,7]. Sporadic outbreaks occur in several species, including the army cutworm (*Euxoa auxiliaris* Grote) [8], bertha armyworm (*Mamestra configurata* Walker) [4], the true armyworm (*Mythimna unipuncta* [Haworth]) and the glassy cutworm (*Apamea devastator* [Brace]) [9,10]. Within Canadian Prairie agroecosystems, the bertha armyworm, the redbacked cutworm, *Euxoa ochrogaster* (Guenée), and the pale western cutworm, *Agrotis orthogonia* Morrison, are the most common species with localized outbreaks in canola and cereal crops [11,12]. Infestation by two or more species can co-occur in the same field [13]. Systematic monitoring of cutworm and armyworm populations across population phases is needed to detect and predict population surges. The foundation of Integrated Pest Management programs is efficient sampling to detect changes in population densities of multiple pests to prevent them from reaching economic injury levels [14].

Female-produced sex pheromones have been identified for most cutworm and armyworm pest species found in the Canadian Prairies [15]. Monitoring programs using synthetic sex pheromone-baited traps were implemented across the region in the 1980s; however, these programs were not widely adopted because moth trap catch did not reflect crop damage, likely because moths were drawn in to sex pheromone traps from long distances [13,16]. Furthermore, pheromone-based monitoring programs require individual traps for each species, which makes monitoring several pests costly and time consuming. Lastly, there is evidence for pollinator by-catch in lepidopteran sex pheromone-baited traps [17,18,19,20]. There are no reliable tools to monitor variation in density of most cutworm adult pest species in the Canadian Prairies. 

Although multiple cues mediate plant–insect interactions, olfaction is perhaps the primary mechanism employed by moths for host selection [21,22]. Cutworm and armyworm moths, like many Lepidoptera, use host plant volatiles for orientation toward food sources, and females may also use these volatiles to select oviposition sites [23]. Furthermore, insects are sensitive to cues produced by microbes associated with their food sources and oviposition sites, referred to as microbial volatile organic compounds (MVOC) [24]. The MVOC hypothesis states that microbial emissions serve as semiochemicals that provide cues regarding suitability and nutritional quality of hosts [24]. Microbes present in floral nectars and fruits produce MVOCs [25], which in combination with floral volatiles can act synergistically to attract lepidopteran herbivores to their hosts. For example, the chemical mixture of acetic acid, a by-product from fermented sugar, and phenylacetaldehyde, a floral volatile, attracts two Noctuidae: the alfalfa looper, *Autographa californica* (Speyer), and the armyworm *Spodoptera albula* (Walker) to baited traps [26]. 

Food-based semiochemicals could be used to monitor multiple cutworm and armyworm species using a single lure, as these cues attract both sexes of multiple species of moths [27]. Overall, food-based semiochemicals are classified into three groups: host plant volatiles, floral volatiles and MVOCs from fermented sugar. Few host plant volatile lures are commercially available to monitor moth pest flight activity or for pest control in attract-and-kill formulations [28,29]. Host plant volatiles may not be important cues for generalist pests, such as the redbacked cutworm or the pale western cutworm, as females of both species oviposit in loose dry soil under crop stubble or in fallow fields rather than on live plant material [2]. Lures baited with floral volatiles from several plants visited by noctuid moths as adult food sources have been used to monitor populations in field experiments [30,31]. For instance, traps baited with phenylacetaldehyde captured soybean looper moths, *Chrysodeixis includens* (Walker) (Lepidoptera: Noctuidae), in cotton fields [32]. Likewise, traps baited with the floral blend of the butterfly bush (*Buddleja davidii* Franch) (Loganiaceae) captured high numbers of the cabbage looper, *Trichoplusia ni* (Hübner) (Lepidoptera: Noctuidae), and alfalfa looper moths [33]. Floral volatile-baited traps, however, result in high pollinator by-catch and have not been adopted commercially to monitor noctuid moths [34,35].

Fermented sugar baits were some of the first food-based semiochemicals used to monitor diversity of Lepidoptera [36]. Noctuidae, Geometridae, Tortricidae and Pyralidae are the major lepidopteran families attracted to these types of baits [37]. Common MVOCs produced from fermented sugar baits are acetic acid, isoamyl alcohol (3-methyl-1-butanol) and isobutanol (2-methyl-1-propanol) [24,37]. Food-based semiochemical lures based on these volatile compounds attract both sexes of many species of noctuid moths [38]. 

The chemical mixture of acetic acid and 3-methyl-1-butanol (hereinafter AAMB) is attractive to several noctuid pests in multiple cropping systems, including the bertha armyworm [39], the true armyworm [40] and the redbacked cutworm [34]. The objective of this study is to develop a food-based semiochemical lure to monitor the cutworm and armyworm pest complex with a single trap and lure and produce minimal impact on native pollinators. Food-based cues attract both male and female moths from the local area and trap catch may be more reflective of local larval densities than pheromone-baited trap capture [38]. Capture of females in baited traps may provide information on the reproductive status of the females and egg load in monitoring programs [41]. Our approach was to enhance the attractiveness of AAMB lures to the most common cutworm species across the Canadian Prairies, the redbacked cutworm, in canola (*Brassica napus* L.) (Brassicaceae) and spring wheat (*Triticum aestivum* L.) (Poaceae) fields. First, we determined the attractiveness of AAMB-baited traps compared to unbaited traps and the respective sex pheromone-baited traps. Second, we tested the attractiveness of AAMB lures at different release rates and the time of its release from different devices. Third, we measured the attraction of the AAMB lure in combination with additional food-based semiochemicals. Finally, we conducted electrophysiological studies on the redbacked cutworm moth to understand the influence of physiological state on response to food-based semiochemicals.

## 2. Materials and Methods

### 2.1. Study Area

A series of experiments were conducted between 2014 and 2015 in fields located in the Aspen Parkland Ecoregion of Alberta province, Canada. The landscape in the region is characterised by extensive agricultural plains with discontinuous clusters of trembling aspen (*Populus tremuloides* Michx) (Salicaceae) and balsam poplar (*P. balsamifera* L.) trees [42]. Seven sites were selected for moth monitoring across central Alberta, dispersed over an area of approximately 7350 km² throughout five counties. Sites were separated by at least 20 km from other experimental sites. Each site consisted of a canola field paired with a wheat field, separated by at least 500 m. All experiments were conducted at the same seven sites each year. Due to crop rotation practices, a canola field in the first year was rotated to a wheat field in the second year.

### 2.2. Lures

Two types of lures were used in all experiments: synthetic female sex pheromones and custom-made food bait lures. Sex pheromone lures targeting different species of cutworm and armyworm moths were used in different field experiments (Table 1). Sex pheromone blends for each of the target species were prepared and loaded onto pre-extracted red rubber septa, prepared by Contech Enterprise Inc. (Delta, BC, Canada). Food bait lures were prepared in the laboratory following the methods of Landolt et al. [34]. The AAMB lure consisted of acetic acid and 3-methyl-1-butanol in a 50:50 by weight mixture (glacial acetic acid (99.7% purity) Fisher Scientific, Fair Lawn, NJ; 3-methyl-1-butanol (98.5% purity) Sigma Aldrich, St. Louis, MO, USA). 

The AAMB chemical mixture was dispensed into a 15 mL narrow-mouth Nalgene HDPE bottle (Thermo Scientific, Rochester, NY, USA) with two cotton balls inserted at the bottom. Each bottle received 10 mL of AAMB chemical mixture. A 3.0 mm diameter hole drilled in the centre of the bottle cap allowed for release of volatiles.

### 2.3. Monitoring and Moth Identification

Non-saturating green universal moth traps (Unitrap, Contech Enterprise Inc., Delta, BC, Canada) were employed in all experiments. Traps were positioned 1.5 m above ground, spaced 25 m apart in a linear transect positioned approximately 5 m from the field edge. Unitraps were baited with either a sex pheromone or a food bait lure. Sex pheromone lures were placed inside baskets positioned under the roof of the unitrap and replaced every four weeks. Food bait lures were secured to the inside wall of the unitrap buckets with a twist-tie and were replaced every two weeks based on the methods of Landolt et al. [34]. An insecticidal strip of Hercon Vaportape II (10% dichlorvos) (Hercon Environmental, Emigsville, PA) was placed inside the bucket of each trap to kill captured insects. Insecticidal strips were replaced every four weeks. 

Insect trap catch was collected every week in plastic bags, labelled and frozen at –20 °C until it was sorted and identified. In the laboratory, moth trap catch and Hymenoptera by-catch were separated from other arthropods. Moths were separated by sex and pinned. If noctuid moths were in poor condition (i.e., no scales on wings or missing body parts), genitalic dissections were performed following the methods developed by Hardwick [43]. To dissect the genitalia, abdomens were removed from moths and immersed in 1 mL potassium hydroxide solution (10% KOH *w/v*) (Biosev, Frenchtown, NJ, USA) in 1.8 mL glass vials (Fisher Scientific, Fair Lawn, NJ, USA) for 48 h to dissolve organs and fatty tissue. Moth genitalia were spread and mounted on cardstock (2.0 × 0.5 cm) with Euparal mounting medium (Bioquip Products Inc. Rancho Dominguez, CA, USA). Moths were identified to species through wing maculation and/or morphological characters of genitalia following taxonomic keys from *The Moths of America North of Mexico* book series [44,45,46,47,48]. Identifications were verified using comparisons with reference collections at the E. H. Strickland Entomological Museum (University of Alberta, Edmonton, AB, Canada). Pinned moths in the best conditions and mounted genitalia dissections from each identified species were selected as voucher specimens and deposited at the E. H. Strickland Entomological Museum, Department of Biological Sciences, University of Alberta, Edmonton.

Hymenoptera by-catch was categorized into two guilds: (1) pollinators, which grouped honeybees (*Apis mellifera* L.) (Apidae), bumblebees (*Bombus* spp.) (Apidae) and leaf-cutter bees (Megachilidae); and (2) vespids (Vespidae), which grouped the bald-faced hornet (*Dolichovespula maculata* L.), the blackjacket (*Vespula consobrina* Saussure) and the common aerial yellowjacket (*D. arenaria* Fabricius). Results from pollinator by-catch in traps baited with lures for monitoring noctuid pest species were reported by Grocock et al. [17], and thus, they are omitted from the results and discussion of this manuscript.

### 2.4. Experiment 1—Efficacy of AAMB Lures to Monitor Cutworm and Armyworm Moths 

We tested the efficacy of AAMB lures to monitor the flight activity and abundance of target pest species compared to their respective sex pheromone. The target pest species were the redbacked cutworm, bertha armyworm, true armyworm and army cutworm. Unitraps were baited with either a synthetic pheromone lure, AAMB lure or left unbaited as control traps. The six traps were positioned in a linear transect, as described above, in random order in both canola and wheat fields at each of the seven sites. The experiment was conducted from 10 June to 10 October 2014. Sex pheromone-baited traps were deployed in the field according to the flight period of the target moth species (Table 1), while the AAMB- and unbaited traps remained in the field throughout the 17-week sampling period. The target moths were identified to species. Other pest species were grouped as ‘other noctuid pest species’. The remaining captured moths were grouped as non-target Lepidoptera. 

Individual analyses for each target species were conducted on the total number of moths captured in the AAMB-baited traps compared to the respective sex pheromone-baited traps and unbaited traps. Similarly, a separate analysis was conducted on the total number of other noctuid pest species captured in all sex pheromone-, AAMB- and unbaited traps. Lastly, the response of vespids to lures targeting cutworm moths was analyzed comparing the total number captured in the differently baited traps. For all analyses in Experiment 1, crop and lure type were specified as explanatory fixed variables and site as a random block factor.

### 2.5. Experiment 2—Attractiveness of AAMB Lures at Different Release Rates

A second experiment evaluated AAMB lures at different release rates to monitor the most abundant cutworm from Experiment 1, the redbacked cutworm. Ten milliliters of the AAMB chemical mixture were loaded in Nalgene HDPE bottles, as previously described, and replaced every two weeks. Release rate was manipulated by the diameter of holes drilled in the center of the bottle cap. Three release rates were tested: low (1.0 mm), standard (3.0 mm), and high (5.0 mm). Captured moths in traps baited with AAMB lures at different release rates were compared to those in the unbaited control traps. The four traps were positioned in a linear transect in random order in both canola and wheat fields at each of the seven sites. The experiment was conducted from 10 June to 2 October 2014. 

AAMB lures were weighed to the nearest 0.001 g (Balance model: XS105 DualRange, Mettler Toledo) before and after deployment in the field to estimate differences in release rates among treatments. The difference in weight (mg) was divided by the period that lures spent out in the field (14 days) to calculate an average release rate per day (mg/day). Individual analyses were performed on the average release rate per day (mg/day) after each lure retrieval. Crop and release rate treatments were specified as explanatory fixed variables, and site as random block factor in the model.

Captured moths over the 16-week trapping period were examined in two separate analyses, on the total number of noctuid moths captured and on the total number of redbacked cutworm moths captured. Crop, moth sex and release rate treatments were specified as explanatory fixed variables, and site as random factor.

### 2.6. Experiment 3—Attractiveness of AAMB Lure Released from Different Devices 

A third field experiment tested the attractiveness of the AAMB emitted from different release devices to monitor redbacked cutworm moths. Release device treatments included 10 mL of AAMB loaded into (1) a Nalgene HDPE bottle, as previously described, with a 3.0 mm diameter hole in the bottle cap secured within the unitrap bucket; (2) a polyethylene bag (12.5 × 3.0 cm) (Contech Enterprise Inc., Delta, BC, Canada) with cellulose sponge (10.0 × 2.5 cm) (Contech Enterprise Inc., Delta, BC, Canada). Bags were hot sealed with an impulse sealer (Midwest Pacific, Taiwan) and hung from the center of the unitrap lid. A third treatment consisted of a 10 g droplet of an inert matrix, Splat™, prepared by ISCA Technologies Inc. (Riverside, CA, USA) loaded with AAMB (1:1 w:w), secured to the inside of the unitrap baskets. An unbaited trap served as a control. The four traps were positioned in a linear transect in random order only on canola fields at each of the seven sites. The experiment was conducted during the peak flight period of redbacked cutworm moth, from 18 August to 15 September 2015. All release devices were replaced every two weeks. 

Following identification, moth trap catches from release device treatments were summed over the 4-week trapping period. Two individual analyses compared moth trap catch in the variously baited traps, the total number of noctuid moths and total number of redbacked cutworm moths. Moth sex and release device treatment were specified as explanatory fixed variables and site as random factor.

### 2.7. Experiment 4—Augmentation of AAMB Lures with Additional Food-Based Semiochemicals 

Experiment 4 evaluated the addition of other food-based semiochemicals to enhance the attraction of the AAMB lure to redbacked cutworm moths. The tested chemicals were alcohol from fermented sugar bait by-products, 2-methyl-1-propanol (hereinafter MP) (>99% purity) (Acros Organics, Fair Lawn, NJ, USA), and a floral volatile, phenylacetaldehyde (hereinafter PAA) (>98% purity) (Acros Organics, Fair Lawn, NJ, USA). Traps were baited with AAMB alone, AAMB+MP, AAMB+PAA, AAMB+MP+PAA, and an unbaited trap served as control. All lures were prepared in the laboratory in equal proportions by weight mixture. Ten milliliters of the chemical mixtures were loaded in Nalgene HDPE bottles, as previously described, with a 3.0 mm diameter hole in the bottle cap for release of the volatile chemical mixture. Bottles were secured to the inside wall of the unitrap bucket with a twist tie and were replaced every two weeks. In addition to the different food bait lures, sex pheromone-baited traps targeting redbacked cutworm moth, bertha armyworm moth, pale western cutworm moth and true armyworm moth were deployed to ensure target moths were present in the field at the time of the experiment (Table 1). The nine baited traps were positioned in a linear transect in random order in both canola and wheat fields at the seven sites. The experiment was conducted from 22 June to 15 September 2015.

Following moth identification, trap catch of the target species in the sex pheromone-baited traps was summed over the 12-week trapping period and analyzed independently for each species. A separate analysis was conducted on the total number of redbacked cutworm moths caught compared with trap catches in the traps baited with the different food bait lures. Lastly, vespid by-catch was analyzed comparing the total number captured in baited traps.

### 2.8. Experiment 5—Electrophysiological Response of Redbacked Cutworm Moths to Food Bait Volatiles

The antennal response plasticity of redbacked cutworm moths to feeding attractants volatiles was evaluated for moths in different physiological conditions through electroantennogram recordings. The sex and feeding status (starved or fed) of moths served as physiological treatments. Redbacked cutworm moths were obtained from a laboratory colony maintained on a pinto-based meridic diet [49] under controlled conditions in growth chambers (Intellus Environmental Controller, Percival Scientific, Perry, IA, USA) at 21 °C and a photoperiod of 16:8 (light: dark). Recently enclosed moths were housed individually in 1000 mL plastic containers with either water or 10% sugar solution. Moths were separated by sex and housed in different growth chambers to avoid exposure of the males to female sex pheromone. Electroantennogram recordings were performed on 10 male and 10 female moths in each feeding group (n = 10 per treatment combination). Electroantennogram recordings were performed on RBC moths past the pre-oviposition period. Moths were 9–10 days old when EAG recordings were performed.

The feeding attractant volatiles presented to redbacked cutworm moth antennae were acetic acid (AA) (99.7% purity) (Fisher, Fair Lawn, NJ, USA), 3-methyl-1-butanol (MB) (98.5% purity) (Sigma Aldrich, St. Louis, MO, USA) and phenylacetaldehyde (PAA) (98% purity) (Acros Organics, Fair Lawn, NJ, USA). The chemicals were serially diluted in HPLC grade hexane (Fisher, Fair Lawn, NJ, USA) to obtain six concentrations (μg/μL): 0.001, 0.01, 0.1, 1.0, 10.0 and 100.0. For each dilution, 50 μL was dispensed on a filter paper strip (0.2 × 7 cm) (Whatman^®^ qualitative filter paper, Grade 1), placed within a disposable Pasteur pipette (14.6 cm, borosilicate glass, Fisher, Fair Lawn, NJ, USA), and allowed to evaporate in the fume hood for 30 min. In addition, 50 μL of hexane and a common plant volatile, (*E*)-2-hexenal (1 μg/μL) (>95% purity) (Aldrich Chemical Co., Milwaukee, WI, USA), were also dispensed on filter paper strips to serve as control and standard, respectively. 

The electroantennogram system consists of an IDAC-02 data acquisition controller system, a Syntech EAG probe (Type PRG-2, internal gain 10X), and EAG 2000 software (Syntech, Hilversum, The Netherlands). Moths were chilled at 4 °C for five min before the right antenna was excised and attached to a stainless steel antenna holder using Spectra 360 conductive gel (Parker Laboratories, Orange, NJ, USA). Carbon-filtered and humidified air, from a Syntech CS-55 stimulus controller, flowed at 50 mL/min over each mounted antenna. Stimulus puffs were triggered by hand via the stimulus controller with pulse duration of 0.2 s and a flow of 10 mL/s. The three compounds were tested on each antenna in the same sequential order: First AA, followed by MB and, lastly, PAA. The stimuli were applied to each antenna once per min in ascending order of concentration, separated by the standard (i.e., hexane, plant volatile, 0.001 μg/μL tested compound, plant volatile, 0.01 μg/μL tested compound, plant volatile, 0.1 μg/μL tested compound, plant volatile, 1.0 μg/μL tested compound, plant volatile, 10.0 μg/μL tested compound, plant volatile, 100.0 μg/μL tested compound, plant volatile). Stimuli were replaced every three h. Independent analyses were conducted for each of the feeding attractant compounds tested. 

### 2.9. Statistical Analyses

For all moth monitoring field experiments (Experiments 1–4), moth trap catch data were analyzed using generalized linear mixed models with Poisson family distribution due to properties of count data, which are bounded to zero, and non-normality of errors [50]. A negative binomial family distribution was specified in models instead of Poisson when trap catch data was over-dispersed [51]. Models were fitted as ‘full models’ at first, in which the fixed component of the models included the main effect of all relevant explanatory variables and all possible interactions. Generalized linear mixed models were analyzed with the ‘glmer’ command for Poisson distribution or ‘glmer.nb’ command for negative binomial distribution in the R package ‘lme4’ v.1.1-17 [52].

For the average release rate per day (mg/day) (Experiment 2) and electroantennogram responses (Experiment 5), data were tested for normality and heteroscedasticity using visual techniques and Shapiro–Wilk test. The difference in release rates among the lures in Experiment 2 was compared with a linear mixed model (Gaussian distribution) with the ‘lme’ command in the R package ‘nlme’ v.3.1-117 [53]. The average release rate per day (mg/day) was square root transformed for normality.

Electroantennogram responses in Experiment 5 were natural logarithm-transformed [ln(x+1)] to meet assumptions of normality. Electroantennogram responses were analyzed in a linear mixed model with random intercept and slope to account for the repeated measures on the same moth antenna. Moth sex, feeding status and dose of the stimulus were specified as explanatory fixed variables. Dose of the stimulus was also specified as the random intercept, and the antenna identification number was considered as the random slope (~Dose|Antenna ID).

For all statistical analyses, model simplification was performed in step-wise a posteriori procedure by removing non-significant interaction terms and comparing nested models through likelihood ratio chi-square test with the ‘anova’ command in R package ‘car’ v.3.0-0 [54]. The optimal model was selected using Akaike’s information criterion (AIC). Test statistic values, degree of freedom numbers and p-values were obtained from the ‘Anova’ function in R package ‘car’ v.3.0-0 [54]. The ‘Anova’ function produces analysis of variance tables from models created by ‘lme’, ‘glmer’ or ‘glmer.nb’ commands. Wald chi-square (Wald χ^2^) tests are calculated for linear mixed models and likelihood ratio chi-square (LR χ^2^) are calculated for generalized linear mixed models. Means comparison for all experiments was performed using Tukey method (α = 0.05) with ‘lsmeans’ package v.2.17 [55]. All statistical analyses were conducted using the freely available statistical package ‘R v.3.5.0’ in ‘RStudio v0.98.’ (http://www.rstudio.com accessed on 29 May 2018).

## 3. Results

### 3.1. Experiment 1—Efficacy of AAMB Lures to Monitor Cutworm and Armyworm Moths 

**Figure 1:**
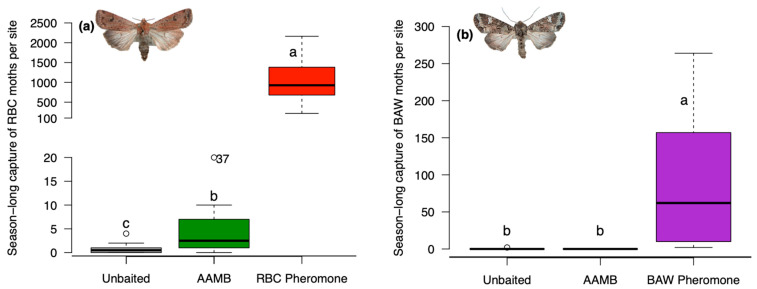
Experiment 1—box-and-whisker plot of the season-long capture of targeted moths in baited traps at seven sites in central Alberta, Canada: (**a**) redbacked cutworm (RBC); (**b**) bertha armyworm (BAW). Open circles represent points more than 1.5 times the interquartile range. Traps were baited with either a sex pheromone, acetic acid and 3-methyl-1-butanol (AAMB) lure or left unbaited as control trap. There was no difference in moth trap catch between canola or wheat fields. Means comparisons were performed for differences in season-long trap capture. Boxplots marked with different letters are statistically different (Tukey method, α = 0.05). (Open circle beside number 37 is data point out of y-axis range in Figure 1a).

The redbacked cutworm moth was the most abundant pest noctuid species captured across all sites. Peak flight activity occurred from 12 August to 10 September (Figure S1). Baited traps captured similar numbers of redbacked cutworm moths in canola and wheat fields (Wald χ^2^ = 2.02; df = 1; *p* = 0.154). The number of redbacked cutworm moths captured in traps differed by the lure type (Wald χ^2^ = 822.92; df = 2; *p* < 0.001). More moths were captured in traps baited with the sex pheromone lure than in AAMB lure or in unbaited traps. More redbacked cutworm moths were captured in AAMB-baited than unbaited traps (Figure 1a). Although redbacked cutworm pheromone-baited traps had high moth trap catch in 2014, infestations were not reported by farmers in 2015.

Similar numbers of bertha armyworm moths were captured in traps positioned in canola and wheat fields (Wald χ^2^ = 0.10; df = 1; *p* = 0.748). Peak flight activity ranged from 30 June to 22 July (Figure S2). Bertha armyworm moths were only caught in their respective sex pheromone-baited trap, and no moths were detected in AAMB or unbaited traps (Wald χ^2^ =131.13; df = 2; *p* < 0.001) (Figure 1b).

**Figure 2:**
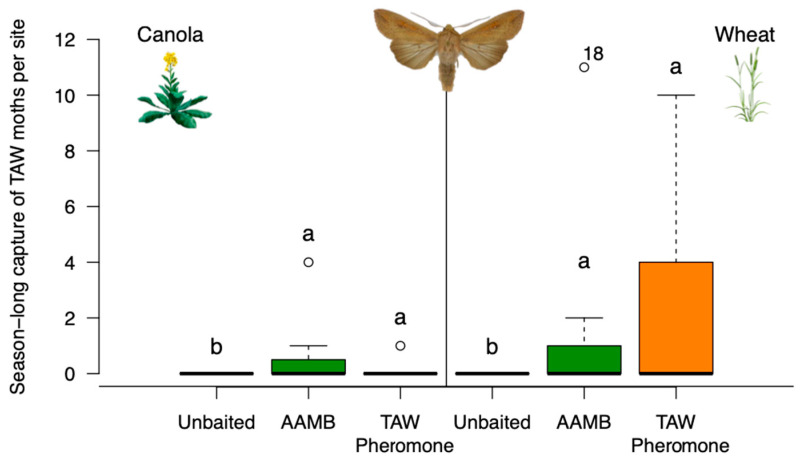
Experiment 1—box-and-whisker plot of the season-long capture of true armyworm (TAW) moths in baited traps at seven sites in central Alberta, Canada. Open circles represent points more than 1.5 times the interquartile range. Traps were baited with either a TAW sex pheromone, AAMB lure or left unbaited as control trap. Means comparisons were performed for differences in trap capture. Boxplots marked with different letters are statistically different (Tukey method, α = 0.05). (Open circle beside number 18 is data point out of y-axis range).

Low numbers of true armyworm moths were captured across all sites, but more moths were recovered from traps in wheat than in canola fields (Wald χ^2^ = 11.57; df = 1; *p* < 0.001). Lure type affected the catch of true armyworm moths (Wald χ^2^ =11.28; df = 2; *p* = 0.003), as traps baited with either pheromone or AAMB similarly caught more moths than unbaited traps did (Figure 2). There were two peaks of true armyworm moths captured in baited traps throughout the season (Figure S3), which suggests two flight periods in the Canadian Prairies. Interestingly, true armyworm moths responded differently to the tested lures depending on the generation of moth. Immigrating moths were attracted to pheromone-baited traps in early summer, while summer generation moths were attracted to AAMB lure-baited traps in early fall.

No army cutworm moths were found in AAMB or unbaited traps, and their respective sex pheromone-baited traps caught extremely low numbers. There was no difference in army cutworm moth trap catch in the variously baited traps.

Eleven percent of moths captured in AAMB-baited traps consisted of the target moth species, while approximately 30% consisted of other noctuid pest species. Other noctuid pest species occurred in higher numbers in the AAMB-baited traps than in the unbaited control traps or traps baited with sex pheromone of the target species, with the exception of the true armyworm sex pheromone trap (Figure S4) (Wald χ^2^ = 175.10; df = 5; *p* < 0.001). Trap catch in the army cutworm sex pheromone-baited traps was dominated by one non-target pest species, the clover cutworm (*Anarta trifolii* [Hugnagel]). Other abundant pest species in the AAMB-baited traps were strawberry cutworm (*Amphipoea interoceanica* Smith), dingy cutworm (*Feltia jaculifera* [Guenée]) and the glassy cutworm (*Apamea devastator* [Brace]) (Table S1).

Traps captured the highest number of vespids in mid-summer, from 15 July to 10 September (Figure S5). Vespids were found in equal numbers in baited traps in canola and wheat fields (Wald χ^2^ = 1.63; df = 1; *p* = 0.200). Vespid by-catch was influenced by lure type (Wald χ^2^ = 40.76; df = 4; *p* < 0.001). Vespid wasps were more significantly attracted to AAMB-baited traps than to the unbaited and sex pheromone-baited traps (Figure S6).

### 3.2. Experiment 2—Attractiveness of AAMB Lures at Different Release Rates

The average release rate (mg/day) fluctuated with treatment across dates. This fluctuation across dates can be attributed to the influence of weather throughout the sampling period. The average release rate differed by treatment on each date (24 Jun: F_2,30_ = 69.84; 08 Jul: F_2,30_ = 50.84; 22 Jul: F_2,30_ = 63.69; 05 Aug: F_2,30_ = 109.32; 18 Aug: F_2,30_ = 113.25; 02 Sep: F_2,30_ = 45.39; 16 Sep: F_2,30_ = 20.11; 02 Oct: F_2,30_ = 18.90) (for all dates: *p*-value < 0.001). The high release rate lures had a higher average release rate than the standard or low release rate lures (Figure S7). The standard lure had a higher average release rate than the low release rate AMMB lure from 24 June to 18 August; however, both treatments had equal average release rates at the end of the season from 2 September to 2 October (Figure S7). The release rates did not differ by trap placement in either canola or wheat fields.

Overall, traps baited with AAMB lures at different release rates captured more noctuid moths than unbaited traps did. AAMB lures captured overall more noctuid moths in wheat fields than in canola (Wald χ^2^ = 51.150; df = 1; *p* < 0.001), and a larger number of male noctuid moths were attracted to AAMB lures than females (Wald χ^2^ = 111.709; df = 1; *p* < 0.001). The effect of the release rate treatment on noctuid moth trap catch depended on interactions with moth sex (release rate · moth sex; Wald χ^2^ = 10.09; df = 3; *p*-value = 0.01) and crop (release rate · crop; Wald χ^2^ = 23.46; df = 3; *p* < 0.001). Male noctuid moths were captured in similar numbers in traps baited with different release rate treatments in canola fields, whereas low release rate lures attracted more male noctuid moths than high-release rate lures in wheat fields (Figure S8). In contrast, female noctuid moth capture did not differ with release rate treatment in wheat fields, but more females were captured in traps baited with the high release rate lures in canola fields (Figure S8).

**Figure 3:**
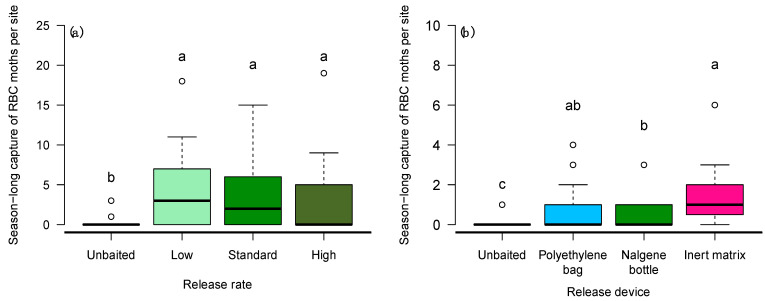
Box-and-whisker plots of the season-long capture of redbacked cutworm (RBC) moths in AAMB-baited traps at seven sites in central Alberta, Canada: (**a**) experiment 2—Different release rates manipulated by the diameter of holes drilled in the centre of the bottle cap: low (1.0 mm), standard (3.0 mm), high (5.0 mm) and an unbaited trap. (**b**) Experiment 3—AAMB lures from different release devices: Polyethylene bag, nalgene bottle, and inert matrix and an unbaited trap. Open circles represent points more than 1.5 times the interquartile range. Means comparisons were performed for differences in moth trap catch among treatments. Boxplots marked with different letters are statistically different (Tukey method, α = 0.05).

Traps baited with AAMB lures captured more redbacked cutworm moths in wheat than in canola fields (Wald χ^2^ = 19.96; df = 1; *p* < 0.001), and more female moths were attracted to AAMB lures than males (Wald χ^2^ = 18.67; df = 1; *p* < 0.001). Traps baited with AAMB lures captured more redbacked cutworm moths than unbaited traps (Wald χ^2^ = 30.827; df = 1; *p* < 0.001); however, the number of moths captured was similar among traps baited with different release rates of AAMB (Figure 3a).

### 3.3. Experiment 3—Attractiveness of AAMB Lure Released from Different Devices 

Overall, traps baited with AAMB lures in different release devices captured more noctuid moths than unbaited traps. The effect of the release device on total noctuid moth trap catch was dependent on moth sex (release device · moth sex; Wald χ^2^ = 9.95; df = 3; *p* = 0.01). Traps baited with the prepared inert matrix captured the highest number of male noctuid moths, while the polyethylene bag and Nalgene bottle lures attracted a similar number of male noctuid moths (Figure S9). Inert matrix-baited traps captured the highest number of female noctuid moths, followed by the polyethylene bag-baited traps and lastly traps baited with Nalgene bottle lures (Figure S9).

Male and female redbacked cutworm moths were captured in similar numbers in traps baited with different release devices (Wald χ^2^ = 2.94; df = 1; *p* = 0.08). Release device had a significant effect on redbacked cutworm moth trap catch (Wald χ^2^ = 30.827; df = 1; *p* < 0.001). The traps baited with the prepared inert matrix captured significantly more moths compared to traps baited with the Nalgene bottles lures, while the polyethylene bag lure captured redbacked cutworm moth in similar numbers to both the other release devices (Figure 3b).

### 3.4. Experiment 4—Augmentation of AAMB Lures with Additional Food-Based Semiochemicals

True armyworm moths were not captured in the traps baited with sex pheromone, food bait lures or in unbaited traps in Experiment 4, and thus, they were considered absent from monitoring sites in 2015. Redbacked cutworm, bertha armyworm and pale western cutworm moths were captured in high numbers in traps baited with their respective sex pheromones in 2015 (Figure S10). More redbacked cutworm and bertha armyworm moths were captured in canola than wheat fields (RBC: Wald χ^2^ = 1038.5; df = 1; *p* < 0.001; BAW: Wald χ^2^ = 170.8; df = 1; *p* < 0.001), while similar numbers of pale western cutworm moths were captured in both crops (Wald χ^2^ = 0.38; df = 1; *p* = 0.536).

**Figure 4:**
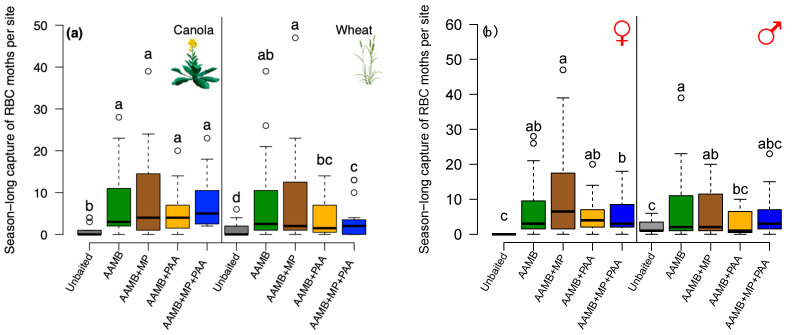
Experiment 4—box-and-whisker plots of the season-long capture of redbacked cutworm (RBC) moths in traps baited with AAMB lures with and without additional chemical compounds. (**a**) By crops: in canola fields (**left** panel) and wheat field (**right** panel); (**b**) by sex: female (**left** panel) and male (**right** panel). Open circles represent points more than 1.5 times the interquartile range. The tested chemicals were alcohols from fermented by-products, 2-methyl-1-propanol (MP), and a floral volatile, phenylacetaldehyde (PAA). Treatments included AAMB alone, AAMB+MP, AAMB+PAA, AAMB+MP+PAA and an unbaited trap that served as the control. Means comparisons were performed for difference in moth trap catch in traps baited with the different food bait lures. Boxplots marked with different letters within each crop type are statistically different (Tukey method, α = 0.05).

The additional chemical compounds added to augment the AAMB lure had a significant effect on redbacked cutworm moth trap catch (Wald χ^2^ = 56.94; df = 4; *p* < 0.001), and this response differed between crops (food bait lure · crop, Wald χ^2^ = 10.74; df = 4; *p* = 0.029). Overall, baited traps captured more moths than unbaited traps. All traps baited with the various food bait lures captured a similar number of redbacked cutworm moths in canola fields (Figure 4a). Conversely, traps baited with food bait lures containing additional alcohol captured more redbacked cutworm moths than food bait lures with floral volatiles in wheat fields. Traps baited with AAMB plus 2-methyl-1-propanol (AAMB+MP) and AAMB alone captured the highest numbers of redbacked cutworm moths, followed by AAMB plus phenylacetaldehyde (AAMB+PAA), and lastly the four-component food bait lure (AAMB+MP+PAA) (Figure 4a).

Response of redbacked cutworm moths to the different food bait lures also varied with sex (food bait lure · moth sex, Wald χ^2^ = 27.61; df = 4; *p* < 0.001). Traps baited with the AAMB+MP lures captured significantly more female moths than traps baited with the four-component lure (AAMB+MP+PAA) did. Female moth trap catch was intermediate in traps baited with the AAMB lure alone and AAMB+PAA (Figure 4b). In contrast, male moth capture was similar in traps baited with AAMB alone, AAMB+MP and AAMB+MP+PAA lures. Traps baited with food bait lures and floral volatiles, AAMB+PAA and the four-component lure, AAMB+MP+PAA, had lower male moth trap catch that did not differ from trap catch in the unbaited control traps (Figure 4b).

The additional chemical compounds also influenced Vespid by-catch. Vespid wasps were found in equal numbers in baited traps in canola and wheat fields (Wald χ^2^ = 0.06; df = 1; *p* = 0.794). Vespid by-catch was influenced by lure type (Wald χ^2^ = 542.0; df = 8; *p* < 0.001). A higher number of vespids were captured in traps baited with food bait lures alone and containing the additional alcohol from fermented by-products, AAMB and AAMB+MP, than in traps with food bait lures and floral volatiles, AAMB+PAA and the four-component lure AAMB+MP+PAA (Figure S11). Sex pheromone traps and unbaited traps had the lowest vespid wasp by-catch.

### 3.5. Experiment 5—Electrophysiological Response of Redbacked Cutworm to Food Bait Volatiles

**Figure 5:**
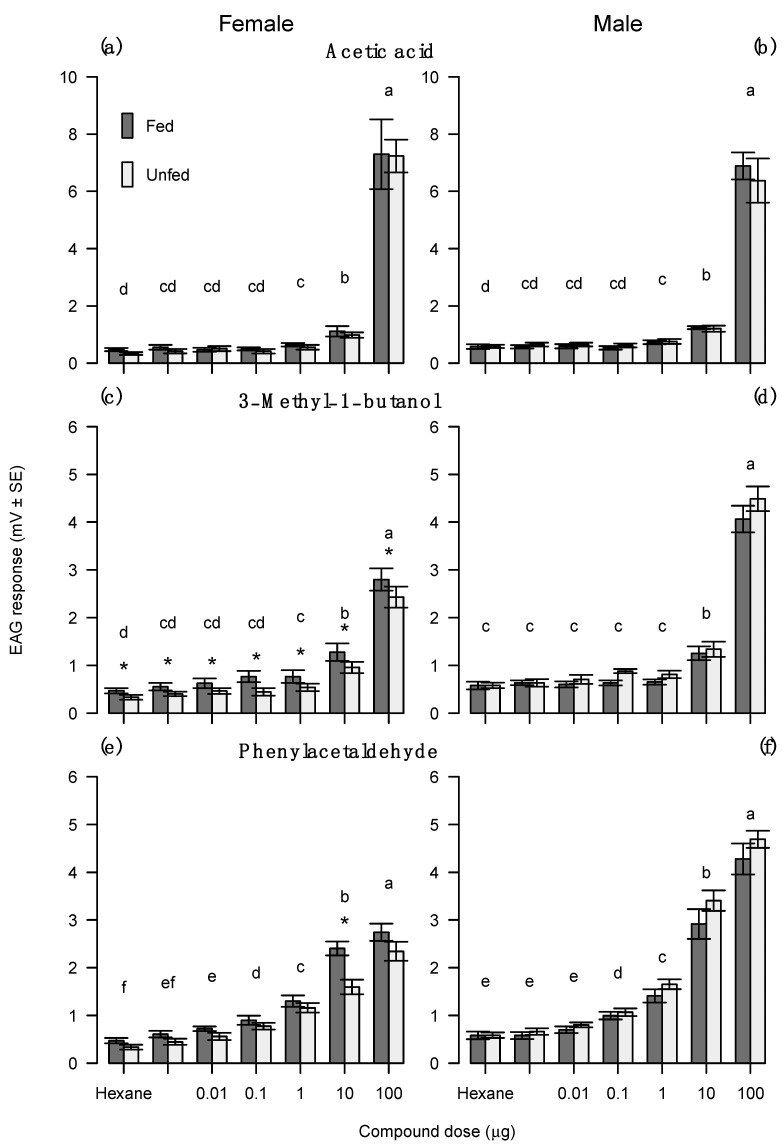
Experiment 6—Average antennal response (mV ± SE) of female (**a**,**c**,**e**) and male (**b**,**d**,**f**) redbacked cutworm moth antennae to feeding attractant volatiles: acetic acid (**a**,**b**), 3-methyl-1-butanol (**c**,**d**) and phenylacetaldehyde (**e**,**f**). Means comparisons were conducted among dose within sex and chemical compound. Bars marked with different letters within each panel are statistically different (Tukey method, α = 0.05). Significant difference between antennal response of fed or unfed moths within dose and chemical compound is represented by (*) (Tukey α = 0.05).

Feeding status did not influence antennal response to acetic acid (F_1,35_ = 0.284, *p* = 0.597). Males had a higher antennal response to acetic acid than females (F_1,35_ = 4.71, *p* = 0.036). Dose had a strong effect on antennal response (F_6,210_ = 562.62, *p* < 0.001). Responses to the lower doses of acetic acid (0.001, 0.01 and 0.1 μg/μL) did not differ from hexane, while significant responses were elicited to the higher doses of acetic acid. The 100.0 μg/μL dose had the highest antennal response, followed by 10.0 and 1.0 μg/μL (Figure 5a,b).

The influence of feeding status on antennal response to 3-methyl-1-butanol was dependent on moth sex (feeding · sex, F_2,35_ = 6.52, *p*-value = 0.015). There were no differences in antennal response between fed or unfed male moths, regardless of the dose (Figure 5d). Fed females had higher antennal responses than unfed females, regardless of the dose (Figure 5c). Dose had a strong effect on antennal response (F_6,210_ = 346.94, *p* < 0.001), and this effect was dependent on moth sex (dose · sex, F_6,210_ = 7.697, *p* < 0.001). Response to the lower doses (0.001, 0.01 and 0.1 μg/μL) was not different from hexane, while higher doses elicited a significant response. Female antennae had the highest response to the 100.0 μg/μL dose, followed by 10.0 and 1.0 μg/μL (Figure 5c). For male antennae, the 100.0 μg/μL dose elicited the highest response, followed by 10.0 μg/μL, but response to the 1.0 μg/μL dose did not differ from hexane (Figure 5d).

Dose had a strong effect on antennal response to phenylacetaldehyde (F_6,210_ = 549.16; *p* < 0.001), and this effect was dependent on moth sex and feeding status (concentration · sex · feeding, F_6,210_ = 2.25, *p* = 0.039). There were no differences in antennal response to the floral volatile between fed or unfed male moths, regardless of the dose (Figure 5f). In contrast, fed females had a higher antennal response than unfed females only at the 10.0 μg/μL dose (Figure 5e). Moth antennae detected phenylacetaldehyde at lower doses compared to acetic acid and 3-methyl-1-butanol. In females, antennal response to the 0.001 μg/μL dose was not different from hexane (Figure 5e). In males, antennal response to doses of 0.001 and 0.01 μg/μL were not different from hexane (Figure 5f). There was a significant dose response with the highest antennal response to 100.0 μg/μL, followed by 10.0 μg/μL, 1.0 μg/μL and 0.1 μg/μL (Figure 5e,f).

## 4. Discussion

This research explored the development of a general food bait lure to attract cutworm and armyworm species with a single trap baited with a single lure in canola and wheat fields in the Canadian Prairies. Field experiments were conducted in canola and wheat fields to evaluate the AAMB lure, a food bait based on microbial volatile compounds from by-products of fermented sugar baits developed by Landolt [39]. 

As expected, traps baited with sex pheromone lures captured a larger number of target moths than any of the food bait lures tested, but the vast majority of moths attracted to sex pheromone-baited traps were males. Male trap catch from pheromone-baited traps may not be representative of the female density. Food bait lures that attract both male and female moths are more suitable tools for monitoring pest species since capture of females might be a better indicator of population density [27]. Capture of females can provide information on the reproductive status of females and egg loading [41]. Furthermore, male moths may be attracted to pheromones from great distances and trap catch may not reflect local population density. Food bait lures may have a smaller attraction radius and capture moths that contribute to the local population density. 

The most abundant pest species were the redbacked cutworm followed by the bertha armyworm in 2014 (Experiment 1) and 2015 (Experiment 4), and the pale western cutworm in 2015 (Experiment 4). True armyworm moths were captured in low numbers in 2014 but were absent in 2015. Although sex pheromones had a high moth trap catch rate of the targeted moths throughout the field seasons, farmers did not report cutworm damage in the following growing season. Noctuid moths are strong flyers and males may be attracted to sex pheromone-baited traps over long distances, and thus, moth trap catch may not reflect the local population density. Pale western cutworm male moths have shown a maximum flight distance of 24 km in flight mill experiments, whereas female moths flew 5 km [56]. Male noctuid moths can disperse over longer distances than females to find a mate. For instance, mark–recapture experiments with sex pheromone-baited traps of the tobacco budworm, *Heliothis virescens* (Fabricious) (Lepidoptera: Noctuidae), showed that moths can disperse up to 30 km from the release point [57]. Furthermore, sex pheromones attract male moths only, and thus, monitoring results may not be representative of female density. Redbacked cutworm male moth trap capture in sex pheromone-baited traps showed no relationship with larval densities in alfalfa fields in Manitoba, Canada [13]. 

Although food baits had a lower moth trap catch than sex pheromone-baited traps, AAMB lures attracted several cutworm and armyworm species, including the target species, the redbacked cutworm and true armyworm. Pale western cutworm and bertha armyworm moths were not captured in AAMB lure-baited traps. Army cutworm attraction to AAMB lures could not be determined since this species was not present at monitoring sites in 2014. It remains to be tested if numbers of target moth species captured in AAMB-baited traps represent local population densities.

Traps baited with the AAMB lures captured redbacked cutworm moths throughout their flight period. The peak flight activity of the redbacked cutworm recorded from traps baited with AAMB lures followed the same pattern as the trap catch in the sex pheromone-baited traps, and thus, the AAMB lure is a potential tool for monitoring the flight activity of this species. For true armyworm, however, only the summer generation moths can be monitored with traps baited with the AAMB lure. Moths from the immigrating generation were captured with sex pheromone but not AAMB lure-baited traps, while summer generation moths were attracted to the AAMB lure but not sex pheromone lures. Differences in response to sex pheromone lures between true armyworm generations has been observed in field experiments [6], in which immigrating moths were captured in sex pheromone-baited and light traps in early summer, while summer generation moths were captured only in light traps in early fall but not in pheromone traps. Sexual maturation in the summer generation moths is delayed under short-day and low-temperature conditions of early fall [58]. These cues induce physiological and behavioural changes in summer generation moths to undertake a southern migration from deteriorating habitats in northern latitudes [6], and therefore, male moths do not respond to sex pheromone lures. 

The true armyworm does not overwinter in Canada and infestations result from moths immigrating in the early summer [59]. The immigrating moths mate and produce a summer generation in early fall. True armyworm may have a plasticity in response to food-based semiochemicals between generations. Our results indicate that moths making the southern migration in the fall are tuned to respond to food cues. Variation in attraction to semiochemicals by insects in different physiological states has been reported in several moth species. For instance, the response of female *Caloptilia fraxinella* (Ely) (Lepidoptera: Gracillaridae) moths to host plant volatiles is higher when they are reproductively active than when females are in reproductive diapause [60]. Similarly, females of the cotton leafworm moth, *Spodoptera littoralis* (Boisduval) (Lepidpotera: Noctuidae), are more attracted to host plant volatiles than to floral volatiles after mating [61]. The true armyworm immigrating generation that flies north may respond to cues for mate finding or oviposition host selection in early summer, while the summer generation moths may have a higher response to food-based semiochemicals to locate food resources prior to southern migration.

In an attempt to enhance the attractiveness of the AAMB lure to target noctuid species, especially the redbacked cutworm moth, different AAMB release rates and release devices were tested. Differences in response between male and female noctuid moths to varying release rates depended on the crops where traps were deployed. It is possible that variation in response is influenced by host plant volatiles from crops in the background where baited traps were positioned. The response of diamondback moths, *Plutella xylostella* L. (Lepidoptera: Plutellidae), to sex pheromone-baited traps is enhanced when combined with green leaf volatiles if traps are deployed in cabbage fields [62] but not in canola fields [63]. Similarly, pea leaf weevil (*Sitona lineatus* L.) (Coleoptera: Curculionidae) has a higher response to semiochemical lures with host plant volatiles in the fall when pea plants are beginning to senescence and crops are harvested than in the spring when crops are at the vegetative growth stage and produce more host plant volatiles that may mask the host volatiles released from baited traps [64]. Overall, baited traps deployed in wheat fields captured more noctuid moths than baited traps in canola fields did. Acetic acid is one of the most prominent volatile organic compounds emitted by canola plants at the flowering stage [65], whereas acetic acid is not part of the volatile profile in wheat plants [66,67]. AAMB lures at varying release rates may be more apparent to target moths in wheat than canola fields. 

Among the different release devices tested, the AAMB chemical mixture incorporated into the inert matrix attracted the most noctuid moths to baited traps, including redbacked cutworm moths. Although release rates of AAMB from the different devices were not measured in this experiment, it is likely that the inert matrix mixture released AAMB at a higher release rate than Nalgene bottles and polyethylene bags. Phenylacetaldehyde incorporated into the wax developed for dispensing semiochemicals has a higher release compared to that of rubber stopper lures, and thus attracts more moths to baited traps [68].

The AAMB lure combined with 2-methyl-1-propanol or phenylacetaldehyde attracts several noctuid pest species in Europe [38], and therefore we hypothesized that the combination could enhance attraction of RBC moths to AAMB lures in Canadian Prairie agroecosystems. An equal number of redbacked cutworm moths were attracted to the different food bait lure types in canola fields, but more moths were captured in traps baited with the AAMB lure with 2-methyl-1-propanol than phenylacetaldehyde in wheat fields. Furthermore, more redbacked cutworm female moths were captured in traps baited with AAMB lures with the additional alcohol from fermented by-products than with AAMB lures with the floral volatile. Several insects rely specifically on microbial volatile organic compounds as cues to locate food sources [21]. For instance, over 90% of moth species captured in traps baited with different sources of fermented sugar baits are noctuids (Noctuidae) [37]. Interestingly, some microbial volatile organic compounds are also constituents of male-produced pheromone signals in noctuid moths. For example, phenylethanol is a component of the male sex pheromone of the flounced chestnut moth, *Agrochola helvola* L. (Lepidoptera: Noctuidae) [69]. Similarly, hairpencils of true armyworm male moths release acetic acid as part of its courtship pheromone blend [70]. Like many noctuid moths, redbacked cutworm is active at night, and therefore moths may rely on microbial volatile organic compounds over floral volatiles to locate food sources. 

Traps baited with AAMB lures captured vespid by-catch, and this attraction is elicited by the short chain alcohol in the food bait chemical mixture [71]. Further studies should evaluate food bait lures with longer chain alcohols to reduce unwanted vespid by-catch. Traps baited with food bait lures based on fermented by-products had a low pollinator by-catch; however, the addition of a floral volatile increased *Bombus* spp. by-catch [17]. Food bait lures based on microbial volatile organic compounds may be especially suitable for monitoring cutworm and armyworm moths because pollinators do not appear to be attracted to these compounds. 

Redbacked cutworm antennae responded to the different food-based semiochemicals in a dose-dependent manner. Higher doses of acetic acid and 3-methyl-1-butanol elicited significant antennal responses, whereas phenylacetaldehyde elicited significant antennal responses at all doses tested. Other moths exhibit dose-dependent antennal responses to host plant volatiles. For example, the host plant volatile, hexan-1-ol, elicits high antennal response at a dose of 0.1 μg in the cotton bollworm moth, *H. armigera* (Hübner) (Lepidoptera: Noctuidae), while response to lower doses (0.0001 to 0.01 μg) does not differ from that to the solvent control [72]. Similarly, phenylacetaldehyde elicits the strongest antennal responses in the lichnis moth, *Hadena bicurris* Hufnagel (Lepidoptera: Noctuidae), at low and high concentrations, while the green leaf volatile, cis-3-hexen-1-yl acetate, elicits antennal recordings only at high concentrations [73]. For the cabbage butterfly, *Pieris rapae* L. (Lepidoptera: Pieridae), antennal response to microbial volatile emissions at high doses may be important for close-range location of food sources [74] rather than the attraction from a distance that was assessed for RBC in the current study. Phenylacetaldehyde elicits higher antennal responses than acetic acid and 3-methyl-1-butanol in redbacked cutworm moth antennae; however, these patterns may be confounded to differences in volatility of the compounds tested. Aromatic aldehydes elicit higher EAG response than alcohols in the cotton bollworm moth [72]. 

The physiological state of insects influences the antennal response to food-based semiochemicals [75]. In the redbacked cutworm moth, males have a higher antennal response to phenylacetaldehyde at high doses than female moths, while both sexes respond similarly to acetic acid and 3-methyl-butanol. Antennal response to acetic acid by true armyworm does not differ between males and females; however, male antennal response to benzaldehyde, a common floral volatile and component of the male-produced true armyworm pheromone blend, is significantly greater than that of females [70]. Several lepidopteran male courtship pheromones are derived from ingested plant compounds, and some of these components are also components of floral scents, such as benzaldehyde or phenylacetaldehyde [76].

In redbacked cutworm moths, feeding status did not influence the antennal response to acetic acid and phenylacetaldehyde in either sex, whereas feeding status did affect the response to 3-methyl-1-butanol in female moths but not in males. Fed unmated female moths displayed a higher antennal response than unfed females. The influence of feeding status on antennal response may be specific to the insect species and the semiochemical cue. For example, fed female *C. fraxinella* have a marginally higher antennal response to the host plant volatiles *(E,E)-α*-farnesene and methyl salicylate compared to unfed females; however, unfed females have a higher antennal response to linalool than fed females [60]. Feeding status, however, highly influences female *C. fraxinella* behavioural response to host plant volatiles, as fed females orient to host plant volatiles more readily than unfed females in wind tunnel experiments [60]. In general, the insect central nervous system seems to be more sensitive to changes in insect physiological state compared to the peripheral system that is less plastic [77]. For example, isothiocyanates stimulate upwind flight in female cabbage moths, *M. brassicae* L. (Lepidoptera: Noctuidae), despite the low antennal response to these compounds [78]. Further studies should evaluate the influence of the tested compounds and the food bait lure blend on redbacked cutworm moth behaviour in wind tunnel studies under controlled conditions. 

## 5. Conclusions

In conclusion, food bait lures based on microbial volatile organic compounds can be further developed to attract redbacked cutworm moths and potentially other cutworm and armyworm pests in field crops, and have a minimum negative effect on native pollinator by-catch [17]. Although food bait lures capture lower numbers of targeted moths compared to sex pheromone-baited traps, both males and females of multiple cutworm species are attracted to AAMB lure. The low number of moths captured in food bait traps may indicate that only moths in the immediate area detect and are attracted to the food bait lures. Future studies should evaluate the attractive radius of these lures and determine whether trap capture represents local population density [79]. Traps baited with the AAMB lure caught more moths in wheat fields than in canola fields, and thus, the background volatile profile of the crop may influence the response to lure. It is possible that microbial volatile organic compounds are more reliable cues for locating food sources than floral volatiles for the redbacked cutworm moths and potentially other noctuid pest species. These types of lures may be more reliable than specific host plant volatiles as several cutworm and armyworm pests are generalists and do not oviposit on host plant tissue, including redbacked and pale western cutworm. The AAMB lures have little impact on pollinator by-catch, but other alcohols should be tested to reduce attraction of vespid wasps to food bait traps. Food bait traps catch a variety of non-pest noctuid moths and entomological expertise is required to separate the species captured.

## Figures and Tables

**Table 1:** Lure composition and deployment schedule for moth monitoring field experiment.

Year	Lure	Components	Ratio	Amount	Time Deployed
2014	Redbacked cutworm (RBC)	Z5-12Ac, Z7-12Ac, Z9-12Ac, Z5-10Ac	200 2 1 1	1000 µg	23 Jun–10 Oct
	Bertha armyworm (BAW)	Z11-16Ac, Z9-14Ac	95 5	500 µg	10 Jun–02 Sep
	True armyworm (TAW)	Z11-16Ac	1	1000 µg	10 Jun–10 Oct
	Army cutworm (ACW)	Z5-14Ac, Z7-14Ac, Z9-14Ac	100 1 10	100 µg	02 Sep–10 Oct
	AAMB	Acetic acid, 3-methyl-1-butanol	1 1	10 mL	10 Jun–10 Oct
	Unbaited control	-	-	-	10 Jun–10 Oct
2015	RBC	Z5-12Ac, Z7-12Ac, Z9-12Ac, Z5-10Ac	200 2 1 1	1000 µg	22 Jun–15 Sept
	BAW	Z11-16Ac, Z9-14Ac	95 5	500 µg	22 Jun–04 Aug
	TAW	Z11-16Ac	1	1000 µg	22 Jun–15 Sept
	Pale western cutworm (PWC)	Z7-12Ac, Z5-12Ac	2 1	500 µg	22 Jun–15 Sept
	AAMB	Acetic acid, 3-methyl-1-butanol	1 1	10 mL	22 Jun–15 Sept
	AAMB+MP	Acetic acid, 3-methyl-1-butanol, 2-methyl-1-propanol	1 1 1	10 mL	22 Jun–15 Sept
	AAMB+PAA	Acetic acid, 3-methyl-1-butanol, phenylacetaldehyde	1 1 1	10 mL	22 Jun–15 Sept
	AAMB+MP+PAA	Acetic acid, 3-methyl-1-butanol, 2-methyl-1-propanol, phenylacetaldehyde	1 1 1 1	10 mL	22 Jun–15 Sept
	Unbaited control	-	-	-	22 Jun–15 Sept

## Data Availability

Data available upon request from Ronald Batallas.

## Supplementary Materials

The following supporting information can be downloaded at: https://www.mdpi.com/article/10.3390/insects14020106/s1, Figure S1: Experiment 1—Average number of redbacked cutworm (RBC) moths (±SE) captured per trap per week; Figure S2: Experiment 1—Average number of bertha armyworm (BAW) moths (±SE) captured per trap per week; Figure S3: Experiment 1—Average number of true armyworm (TAW) moths (±SE) captured per trap per week; Figure S4: Experiment 1—Box-and-whisker plot of the season-long capture of other noctuid pest species in baited traps; Table S1: Average (±SE) number of the season long capture of other noctuid pest species in AAMB baited traps in Experiment 1; Figure S5: Experiment 1—Average number of vespids (±SE) captured per trap per week; Figure S6: Experiment 1—Box-and-whisker plot of the season-long capture of vespids in baited traps; Figure S7: Experiment 2—Average release rate per day (mg/day ± SE) of AAMB lure from Nalgene 10 mL bottle lures with different sized holes in the centre of the bottle cap; Figure S8: Experiment 2—Box-and-whisker plots of the season-long capture of male (top panels) and female (bottom panels) noctuid moths in AAMB-baited traps at different release rates manipulated by the diameter of holes drilled in the centre of the bottle cap; Figure S9: Experiment 3—Box-and-whisker plots of the season-long capture of female (left panel) and male (right panel) noctuid moths in traps baited with AAMB lures from different release devices; Figure S10: Experiment 4—Box-and-whisker plots of the season-long capture of target pest species in their respective sex pheromone-baited traps positioned in canola or wheat fields; Figure S11: Experiment 4—Box-and-whisker plots of the season-long capture of vespid by-catch in traps baited with lures targeting cutworm moths.
