# Supplementary material for: Fermented or Floral? Developing a Generalized Food Bait Lure to Monitor Cutworm and Armyworm Moths (Lepidoptera: Noctuidae) in Field Crops
Source: Insects. 2023 Jan 19;14(2):106. doi: 10.3390/insects14020106 (PMC9959891; doi:10.3390/insects14020106)
Supplement: Supplementary file 1 [file insects-14-00106-s001.zip › insects-2142831-supplementary.pdf]

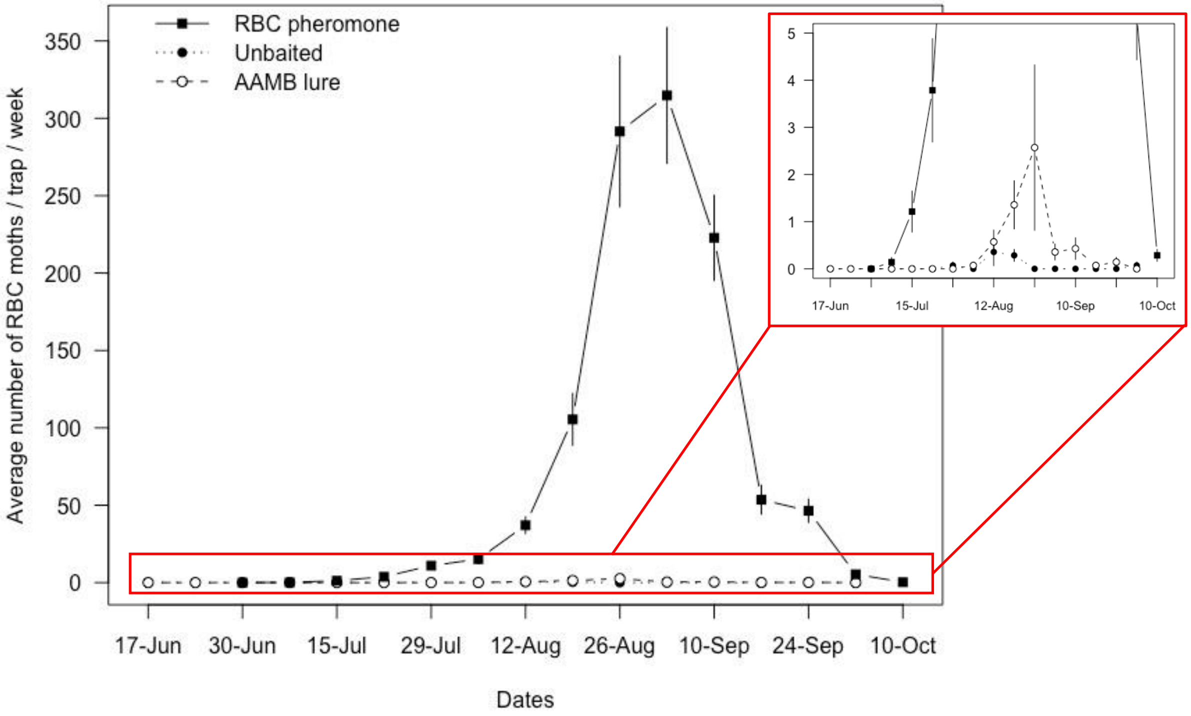

Figure S1. Experiment 1 - Average number of redbacked cutworm (RBC) moths (± SE) captured per trap per week at seven sites in central Alberta, Canada. RBC moths were captured in three differently baited traps: RBC sex pheromone, acetic acid and 3-methyl-1-butanol (AAMB) lure and an unbaited trap.

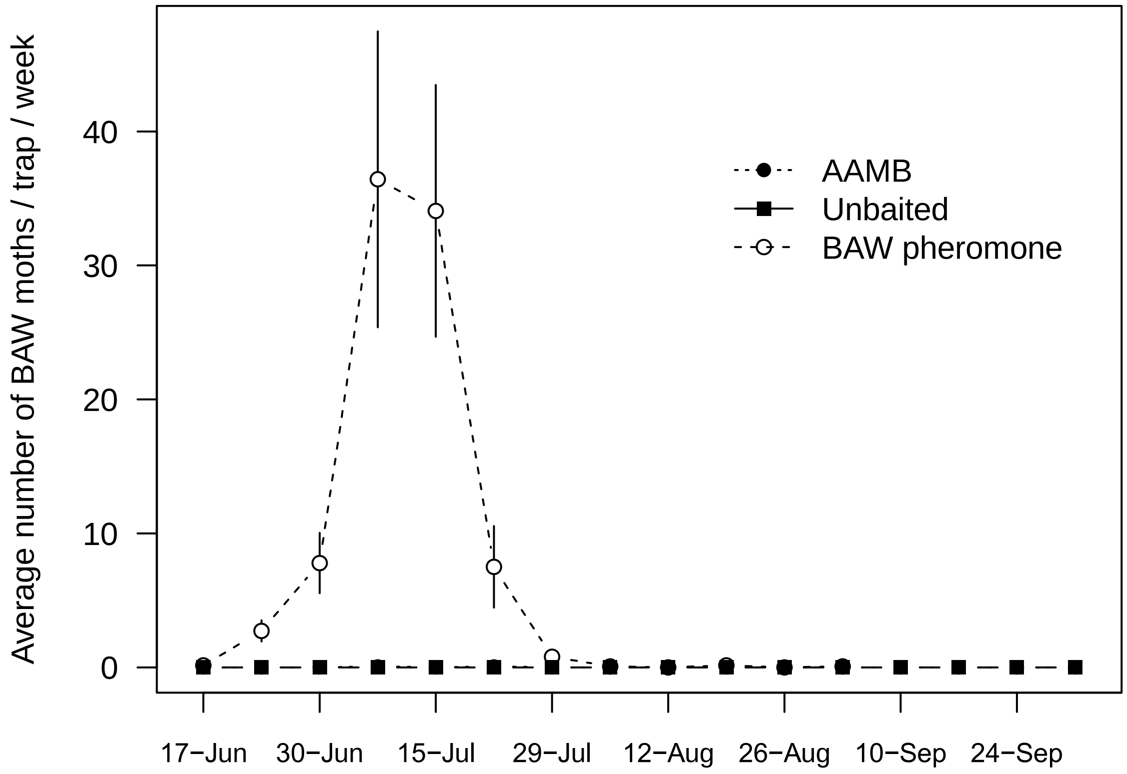

Figure S2. Experiment 1 - Average number of bertha armyworm (BAW) moths (± SE) captured per trap per week at seven sites in central Alberta, Canada. BAW moths were captured in three differently baited traps: BAW sex pheromone, acetic acid and 3-methyl-1-butanol (AAMB) lure and an unbaited trap.

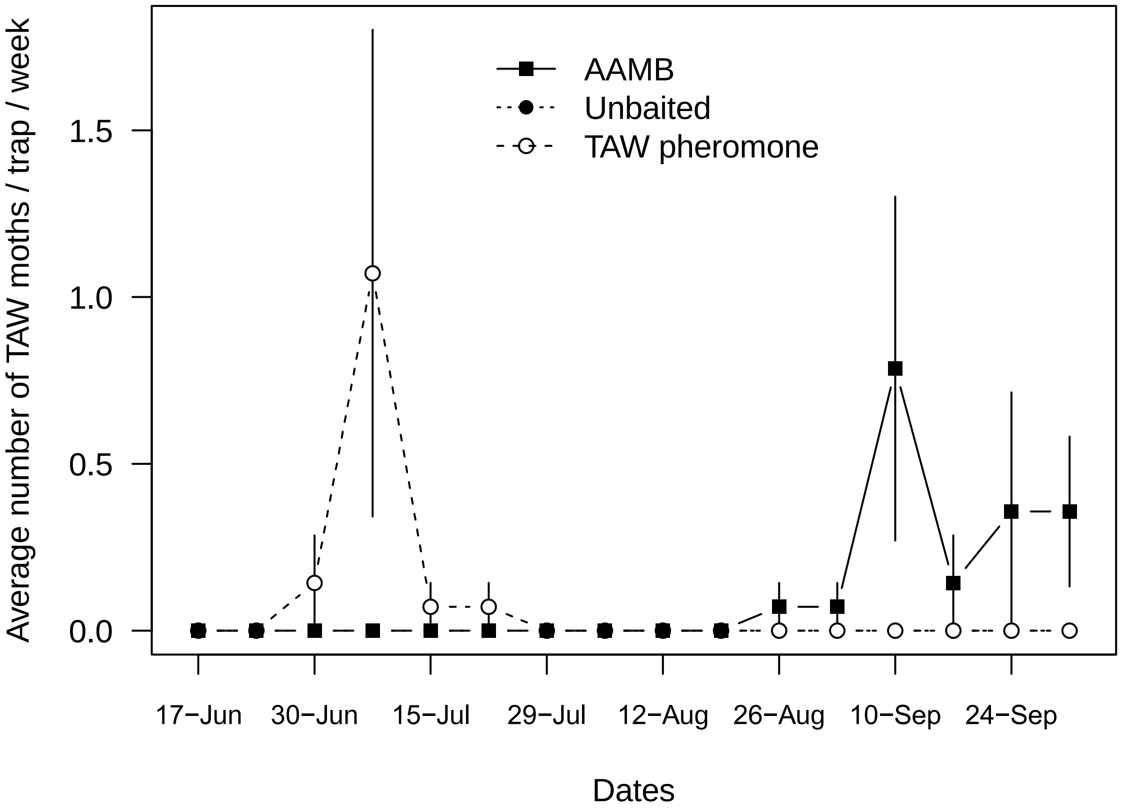

Figure S3. Experiment 1 - Average number of true armyworm (TAW) moths (± SE) captured per trap per week at seven sites in central Alberta, Canada. TAW moths were captured in three differently baited traps: TAW sex pheromone, acetic acid and 3-methyl-1-butanol (AAMB) lure and an unbaited trap.

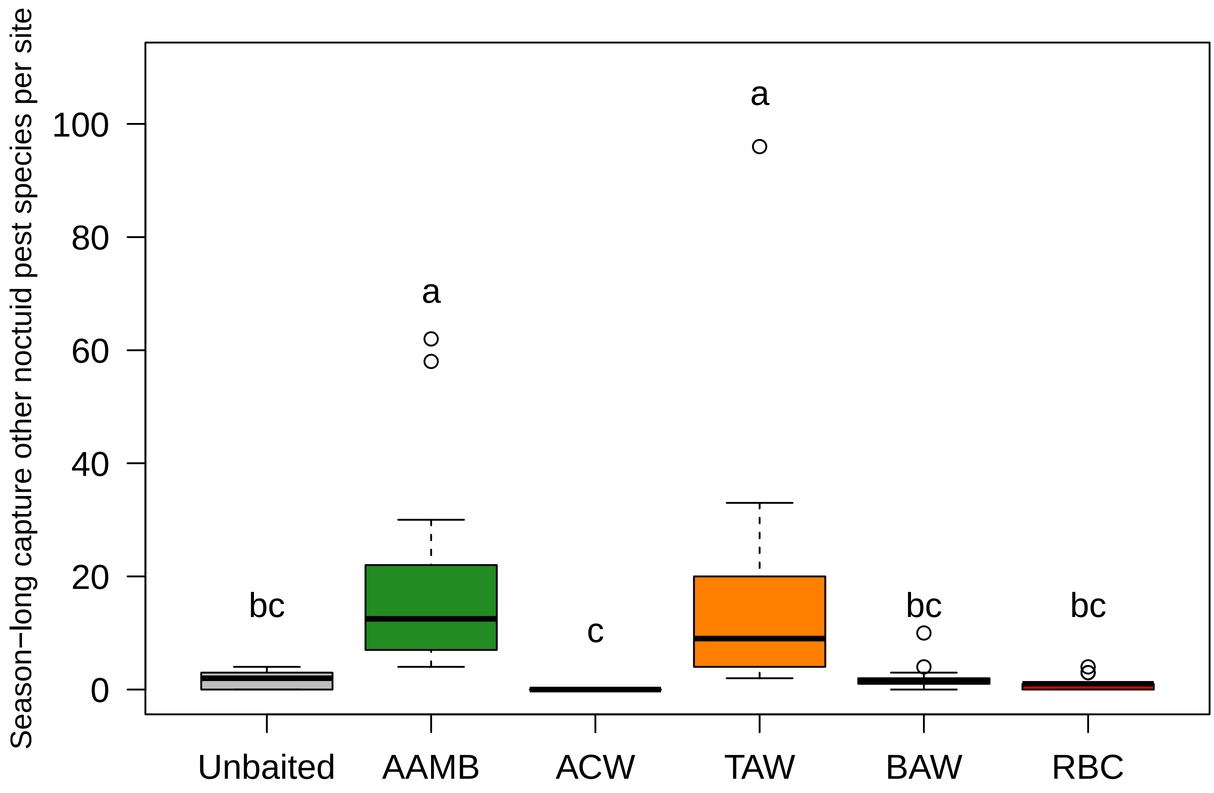

Figure S4. Experiment 1 – Box-and-whisker plot of the season-long capture of other noctuid pest species in baited traps at seven sites in central Alberta, Canada. Traps were baited with either an army cutworm (ACW) sex pheromone, true armyworm (TAW) sex pheromone, bertha armyworm (BAW) sex pheromone, redbacked cutworm (RBC) sex pheromone, AAMB lure or left unbaited as control trap. Means comparisons were performed for differences in trap capture. Boxplots marked with different letters are statistically different (Tukey method, $\alpha = 0.05$).

Table S1. Average (± SE) number of the season long capture of other noctuid pest species in AAMB baited traps in Experiment 1. Seven central Alberta, Canada. Each site consisted of canola field paired with a wheat field.

				Canola		Wheat	
**Tribe**	**Subtribe**	**Scientific name**	**Common name**	**Female**	**Male**	**Female**	**Male**
Noctuini	Agrotina	*Euxoa ochrogaster*	Redbacked cutworm	2.28 ± 1.17	1.28 ± 0.52	4.86 ± 2.99	2.71 ± 2.06
		Euxoa auxiliaris	Army cutworm	0.00 ± 0.00	0.00 ± 0.00	0.00 ± 0.00	0.00 ± 0.00
		Euxoa scandens	White cutworm	0.00 ± 0.00	0.00 ± 0.00	0.00 ± 0.00	0.14 ± 0.14
		Feltia jaculifera	Dingy cutworm	1.14 ± 0.40	2.29 ± 0.42	1.29 ± 0.74	6.42 ± 5.18
		Agrotis venerabilis	Dusky cutworm	0.14 ± 0.14	0.00 ± 0.00	0.14 ± 0.14	0.29 ± 0.18
		Agrotis orthogonia	Pale wester cutworm	0.00 ± 0.00	0.00 ± 0.00	0.00 ± 0.00	0.00 ± 0.00
	Noctuina	*Noctua pronuba*	Winter cutworm	0.14 ± 0.14	0.00 ± 0.00	0.00 ± 0.00	0.14 ± 0.14
Apameini		*Apamea devastator*	Glassy cutworm	0.42 ± 0.30	0.86 ± 0.46	0.57 ± 0.42	2.57 ± 1.04
		Apamea amputatrix	Yellow head cutworm	0.14 ± 0.14	0.00 ± 0.00	0.00 ± 0.00	0.00 ± 0.00
		Amphipoea interoceanica	Strawberry cutworm	2.28 ± 0.89	4.0 ± 2.03	3.14 ± 1.26	8.14 ± 4.1
Hadenini		*Xestia c-nigrum*	Spotted cutworm	0.29 ± 0.18	0.00 ± 0.00	0.00 ± 0.00	0.00 ± 0.00
		Anarta trifolii	Clover cutworm	0.00 ± 0.00	0.00 ± 0.00	0.00 ± 0.00	0.00 ± 0.00
		Dargida procinctus	Olive green cutwom	0.00 ± 0.00	0.00 ± 0.00	0.00 ± 0.00	0.14 ± 0.14
		Mamestra configurata	Bertha armyworm	0.29 ± 0.18	0.14 ± 0.14	0.28 ± 0.18	0.29 ± 0.18
Eriopygini		*Lacinipolia renigera*	Bristly cutworm	0.28 ± 0.18	0.00 ± 0.00	0.14 ± 0.14	0.42 ± 0.20
Tholerini		*Nephelodes minians*	Bronzed cutworm	0.00 ± 0.00	1.26 ± 0.18	0.28 ± 0.28	1.42 ± 0.52
Leucanii		*Mythimna unipuncta*	True armyworm	0.57 ± 0.42	0.14 ± 0.14	1.71 ± 1.41	1.14 ± 1.14

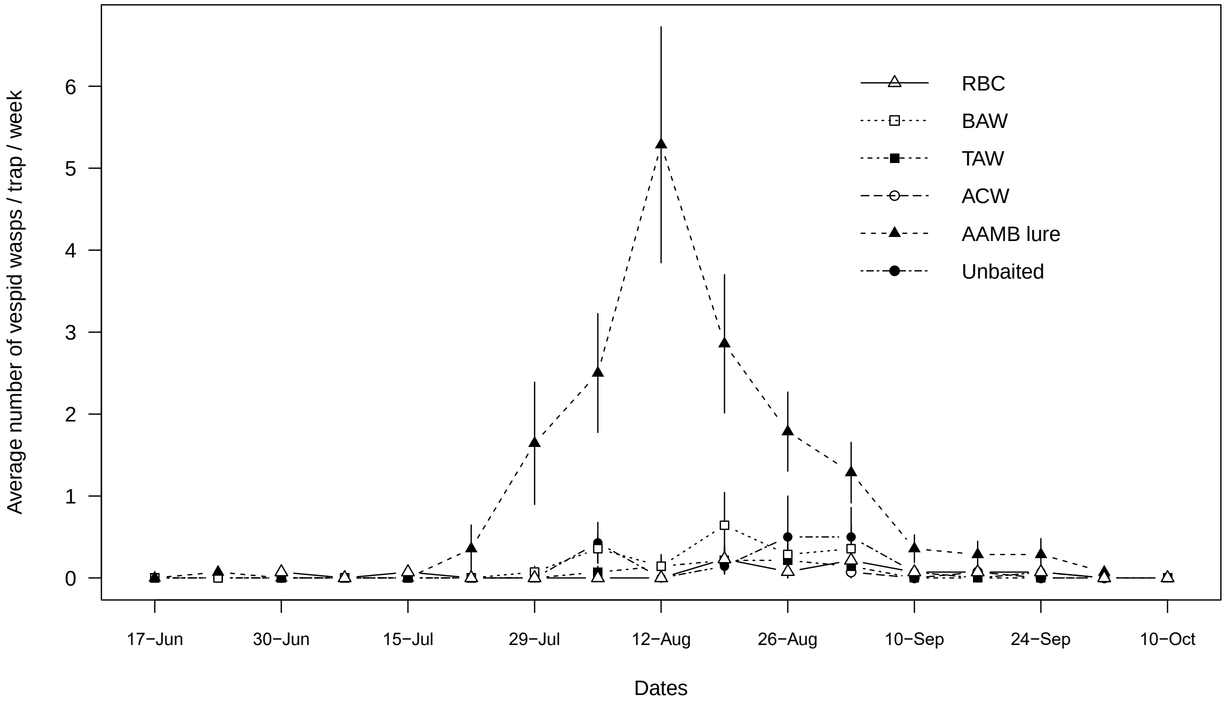

Figure S5. Experiment 1 - Average number of vespids (± SE) captured per trap per week at seven sites in central Alberta, Canada. Vespids were captured in three differently baited traps: army cutworm (ACW) sex pheromone, true armyworm (TAW) sex pheromone, bertha armyworm (BAW) sex pheromone, redbacked cutworm (RBC) sex pheromone, AAMB lure or left unbaited as control trap.

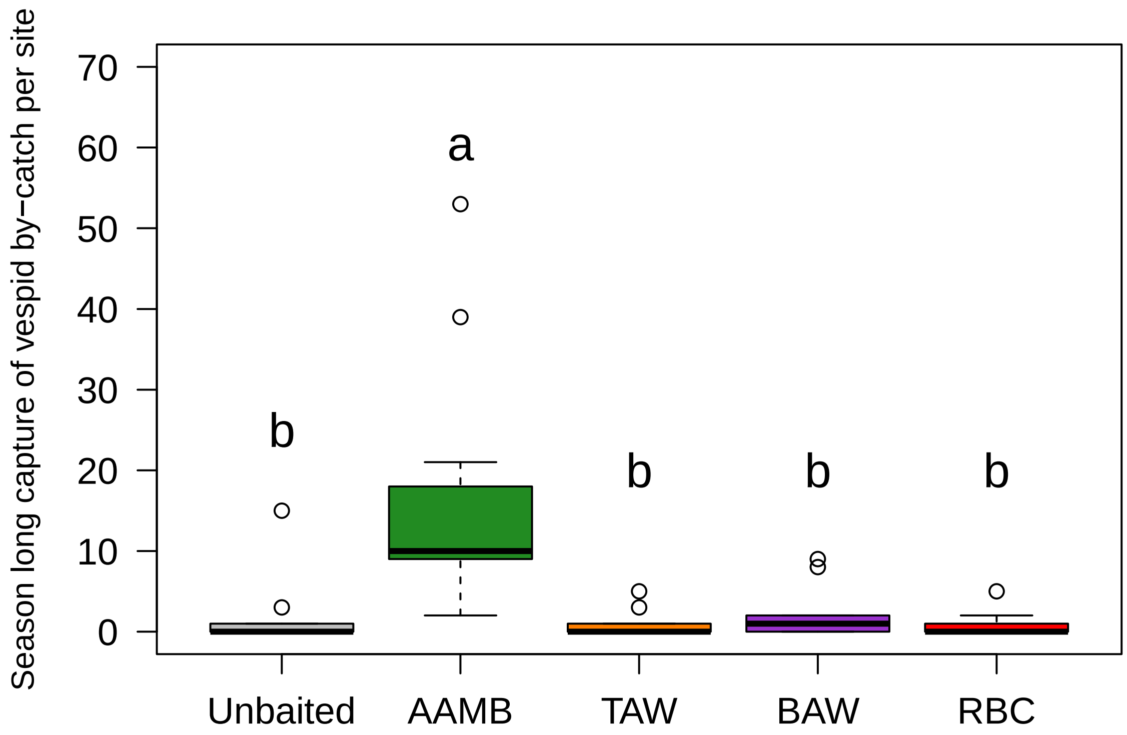

Figure S6. Experiment 1 – Box-and-whisker plot of the season-long capture of vespids in baited traps at seven sites in central Alberta, Canada. Traps were baited with either an army cutworm (ACW) sex pheromone, true armyworm (TAW) sex pheromone, bertha armyworm (BAW) sex pheromone, redbacked cutworm (RBC) sex pheromone, AAMB lure or left unbaited as control trap. Means comparisons were performed for differences in trap capture. Boxplots marked with different letters are statistically different (Tukey method, $\alpha = 0.05$).

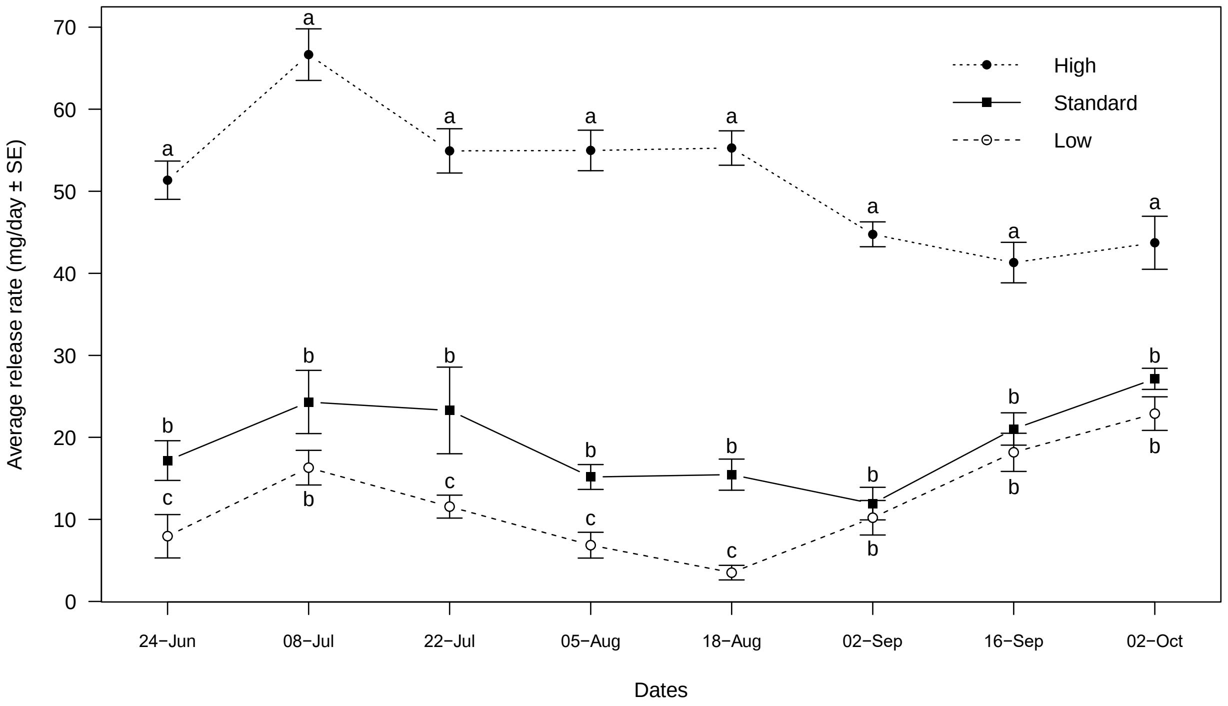

Figure S7. Experiment 2 – Average release rate per day (mg/day ± SE) of AAMB lure from Nalgene 10 mL bottle lures with different sized holes in the centre of the bottle cap. Three release-rates were tested: low (1.0 mm diam. hole in lid), standard (3.0 mm diam. hole in lid), high (5.0 mm diam. hole in lid). Lures were replaced every 2 weeks, at which time individual analyses were performed for AAMB lures retrieved from the field. There was no difference in average release rate of lures positioned in traps in canola or wheat fields. Means comparisons were performed for differences among release rate treatments. Points marked with different letters within date are statistically different (Tukey method, $\alpha = 0.05$).

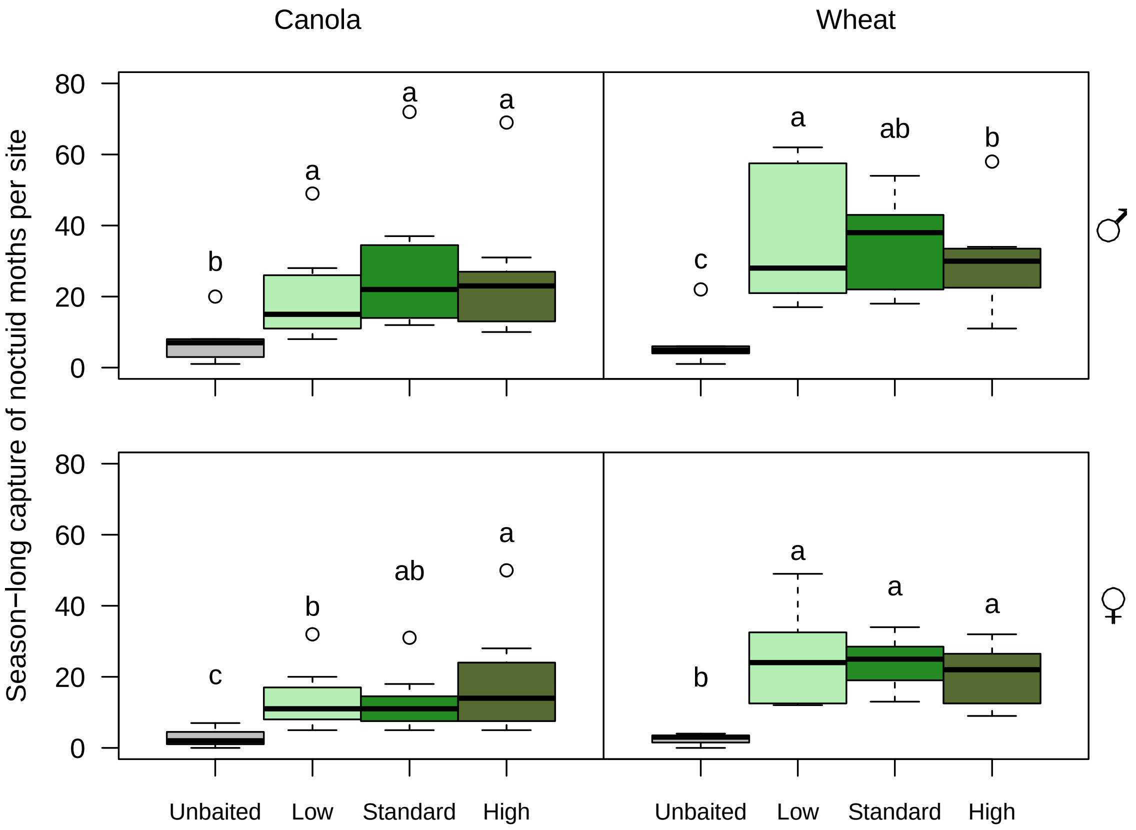

Figure S8. Experiment 2 - Box-and-whisker plots of the season-long capture of male (top panels) and female (bottom panels) noctuid moths in AAMB baited traps at different release rates manipulated by the diameter of holes drilled in the centre of the bottle cap. Three release rates were tested: low (1.0 mm), standard (3.0 mm), high (5.0 mm); and an unbaited trap. Means comparisons were performed for difference in moth trap catch among the treatments by the interaction of crop and sex. Boxplots marked with different letters within moth sex and crop panel are statistically different (Tukey method, $\alpha = 0.05$).

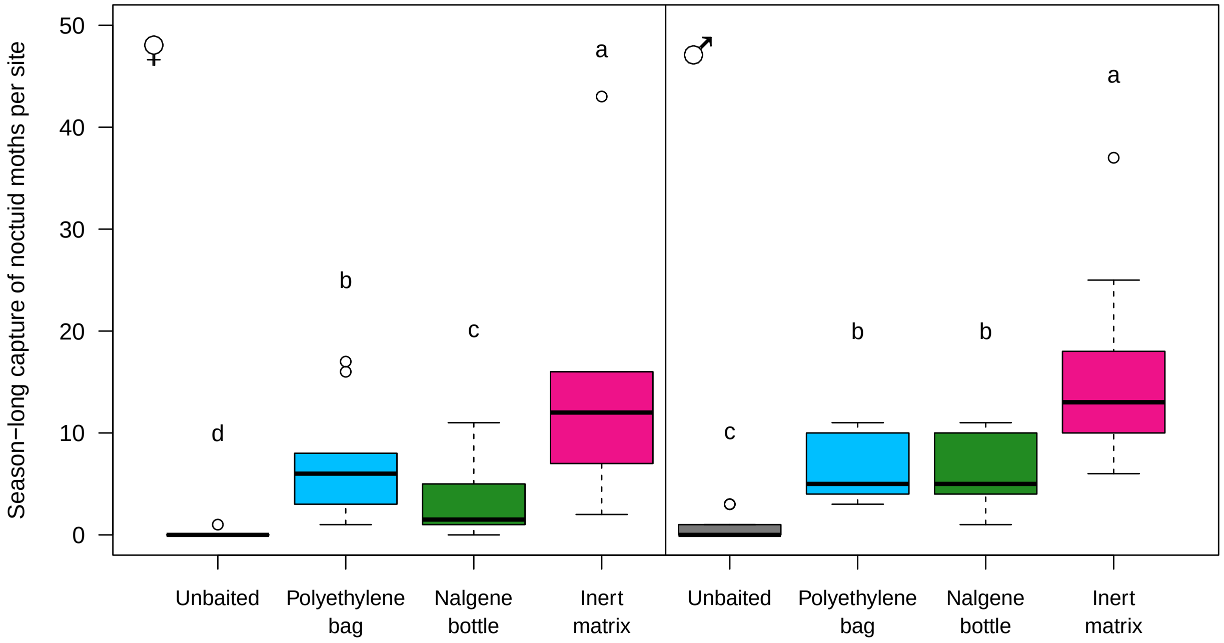

Figure S9. Experiment 3 - Box-and-whisker plots of the season-long capture of female (left panel) and male (right panel) noctuid moths in traps baited with AAMB lures from different release devices. Three release-devices were tested: 10 mL nalgene bottle (Bottle), 10 mL polyethylene bag (Bag) and 10 g splat matrix (Splat); and an unbaited trap. Means comparisons were performed for difference in moth trap catch in traps baited with the different release devices. Boxplots marked with different letters within moth sex panel are statistically different (Tukey method, $\alpha = 0.05$).

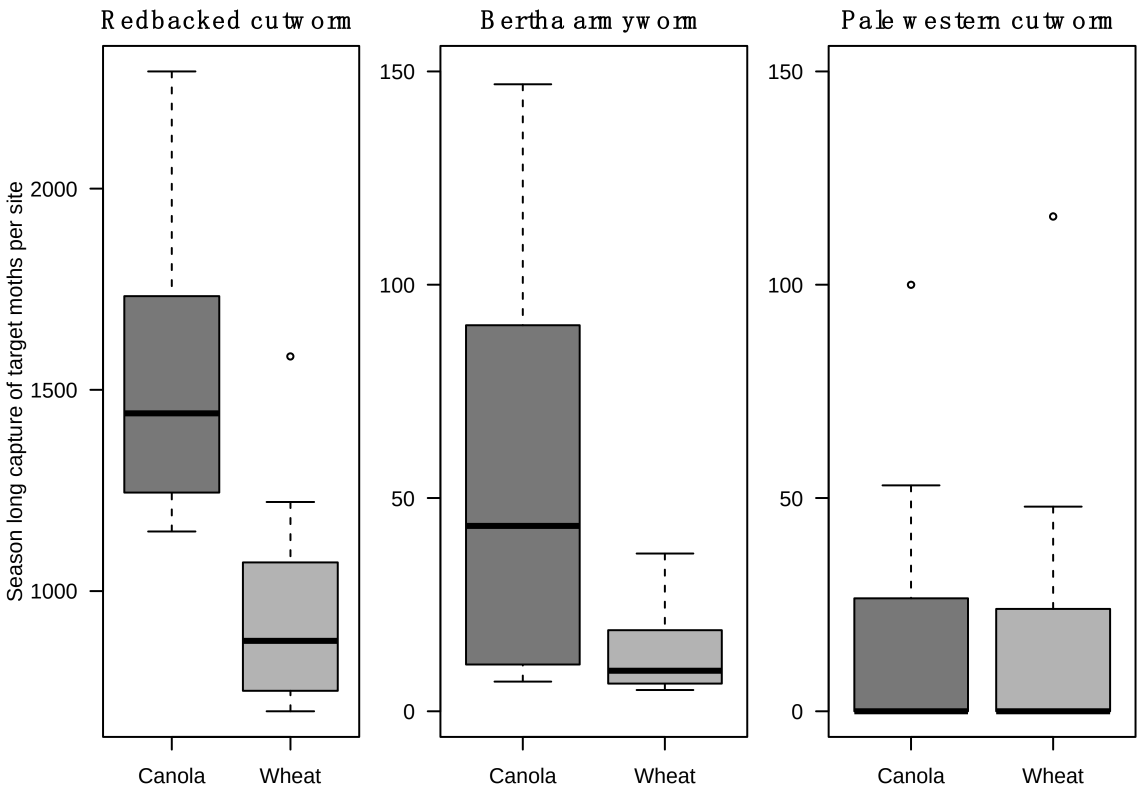

Figure S10. Experiment 4 - Box-and-whisker plots of the season-long capture of target pest species in their respective sex pheromone baited traps positioned in canola or wheat fields at seven sites in central Alberta, Canada.

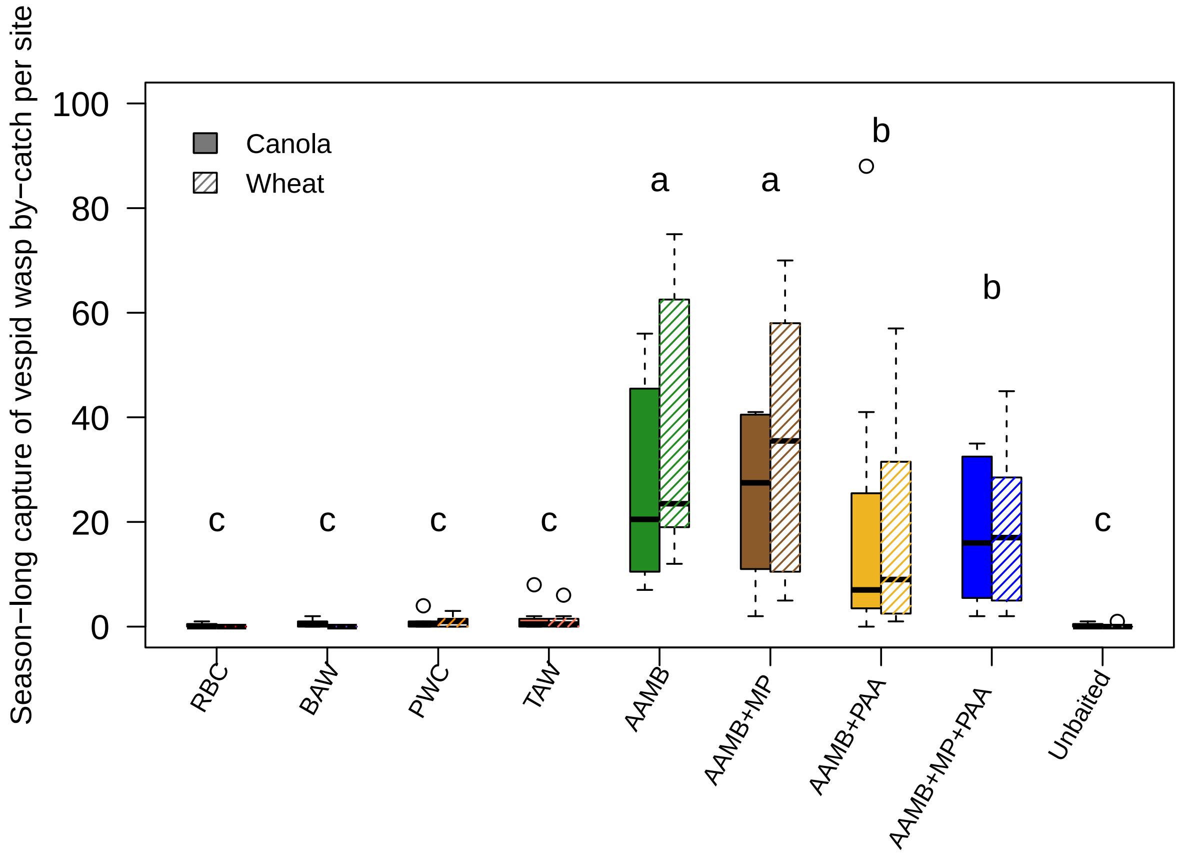

Figure S11. Experiment 4 - Box-and-whisker plots of the season-long capture of vespid by-catch in traps baited with lures targeting cutworm-moths. There was no difference in vespid by-catch in traps positioned in canola and wheat fields. Means comparisons were performed for differences in total trap catch in traps baited with different lure types: redbacked cutworm sex pheromone (RBC), bertha armyworm sex pheromone (BAW), pale western cutworm sex pheromone (PAW), true armyworm sex pheromone (TAW), AAMB alone, AAMB plus 2-methyl-1-propanol (AAMB+MP), AAMB plus phenylacetaldehyde (AAMB+PAA), quaternary lure (AAMB+MP+PAA) and in an unbaited trap that served as control. Boxplots marked with different letters are statistically different (Tukey method, $\alpha = 0.05$)
